# Pathways linking workplace violence and suicidal ideation/non-suicidal self-injury among nurse staff: the mediating role of loneliness and depressive symptoms

**DOI:** 10.1186/s12912-024-02044-2

**Published:** 2024-05-31

**Authors:** Changmian Ding, Zhizhou Duan, Wenqun Luo, Lidan Li, Guizhi Li, Xuehua Li, Linli Xie, Rongrong Yang

**Affiliations:** 1 The Medical Record Management Department, The People’s Hospital of Dehong, Yunnan, China; 2 Preventive health service, Jiangxi provincial people’s Hospital, The First Affiliated Hospital of Nanchang Medical College, Preventive health service, Nanchang, Jiangxi China; 3 Department of Gynecology, Jiangxi Provincial People’s Hospital, The First Affiliated Hospital of Nanchang Medical College, Nanchang, Jiangxi China; 4 The Nursing Department, The People’s Hospital of Dehong, Yunnan, China; 5 Emergency Department, The People’s Hospital of Dehong, Yunnan, China

**Keywords:** Nurse, Workplace violence, Suicidal ideation, Loneliness, Non-suicidal self-injury

## Abstract

**Background:**

Nurses face disproportionately high rates of suicidal ideation and non-suicidal self-injury (NSSI). The role of workplace violence, loneliness, and depressive symptoms in exacerbating these issues is poorly understood. This study aims to explore these relationships to inform interventions for improving nurses’ mental health.

**Methods:**

A cross-sectional study involving 1,774 Chinese nurse staff selected through convenient sampling methods was conducted. Workplace violence, depressive symptoms, and loneliness were assessed using the Chinese versions of the Workplace Violence Scale (WVS), the 9-item Patient Health Questionnaire (PHQ-9), and a three-item loneliness scale, respectively. Participants completed self-report questionnaires anonymously to ensure adherence to ethical standards. Statistical analysis utilized structural equation modeling (SEM) to examine the intricate relationships among variables, thereby elucidating the impact of workplace violence, loneliness, and depressive symptoms on nurses’ suicidal ideation/NSSI outcomes.

**Results:**

Nurse staff 165 (7.8%) were reported different level of suicidal ideation and 139 (7.8%) participants were reported different level of NSSI. And the final model of workplace violence on suicidal ideation shown a good model fit index (CMIN/DF = 3.482 NFI = 0.969 CFI = 0.977 TLI = 0.955 RFI = 0.938, RMSEA = 0.037 SRMR = 0.035). The pathway of workplace violence to loneliness (β = 0.163, *P* < 0.001), the indirect effect of workplace violence on suicidal ideation via loneliness and depressive symptoms were 0.100 (95%CI = 0.085, 0.121), the indirect effect of loneliness on suicidal ideation via depressive symptoms were 0.128 (95%CI = 0.100, 0.158). Similarly, the final model of workplace violence on NSSI shown a good model fit index (CMIN/DF = 3.482 NFI = 0.967 CFI = 0.976 TLI = 0.953 RFI = 0.935, RMSEA = 0.037 SRMR = 0.034), the pathways of workplace violence to NSSI (β = 0.115, *P* < 0.001), the indirect effect of workplace violence on NSSI via loneliness and depressive symptoms were 0.075 (95%CI = 0.055, 0.096), the indirect effect of loneliness on NSSI via depressive symptoms were 0.102 (95%CI = 0.076, 0.130).

**Conclusion:**

Our study unveils the role of workplace violence in nurses’ suicidal ideation and NSSI, mediated by loneliness and depressive symptoms. Interventions targeting workplace violence are crucial for nurses’ well-being, potentially reducing loneliness and depressive symptoms and lowering the risk of suicidal ideation and NSSI. However, further research is needed to explore additional mediators and pathways, employing longitudinal designs to establish causality and develop tailored interventions for nurses affected by workplace violence.

## Introduction

Suicidal ideation and non-suicidal self-injury (NSSI) have been persistent issues in society for a considerable length of time and pose significant public health concerns, placing a heavy burden on individuals, families, communities, and counties [1, 2]. Nursing professionals have been shown to have higher rates of suicidal ideation and NSSI. According to the findings of a study conducted by Chen in Taiwan, 18.3% of nurse staff reported experiencing suicidal thoughts within the past week [3]. Additionally, a survey conducted in Hong Kong revealed that 14.9% of participants had considered suicide within the past year, and 9.3% reported engaging in NSSI within the same time frame [4, 5]. Recent studies have revealed that nurses are more prone to experiencing suicidal ideation and NSSI compared to the general population [4, 6]. The significant occupational stresses, such as heavy workloads and widespread job dissatisfaction, can be attributed to this phenomenon [7, 8]. Additionally, the nature of nursing work can further increase the risk of nurses developing suicidal ideation and NSSI [4, 9]. Understanding the intricate interplay of factors contributing to suicidal ideation and NSSI among nurses is crucial for developing effective interventions.

Depressive symptoms are the most commonly reported mental health difficulties among nurse staff [10] and are considered significant risk factors for NSSI and suicidal ideation [11, 12]. Research has shown that approximately 90% of individuals who die by suicide display symptoms of depression, and depression and other psychiatric disorders contribute to 47–74% of the population risk of suicide [13]. A close link between depressive symptoms and suicidal ideation has been suggested by a recent study conducted by Wu, which found that in 94.2% of cases, depressive symptoms accurately predicted the presence of suicidal ideation [14]. Previous research has also confirmed that depressive symptoms exacerbate NSSI. For example, a longitudinal analysis conducted by Marshall SK et al. found that depressive symptoms predicted NSSI in a one-year follow-up study among adolescents [15]. Similarly, a study of 275 teenagers aged 12 to 17 found that depressive symptoms were a significant risk factor for NSSI in later development [16]. Depressive symptoms have been linked to increased likelihood of engaging in suicidal ideation/NSSI, emphasizing the need for targeted interventions within the nursing community.

Loneliness has been linked to various negative mental health consequences such as depression, suicidal ideation, and NSSI [17–19]. Loneliness has been acknowledged as a significant factor correlated with depressive symptoms, as evidenced by numerous studies [20, 21]. In particular, a meta-analysis of 33 studies conducted on adolescents indicated that the link between depression and loneliness is strongly pronounced, with a large effect size ranging from *r* = 0.55 to 0.60 [22]. A cross-sectional study has indicated that loneliness is one of the most significant factors contributing to suicidal ideation [23]. Furthermore, research has shown that loneliness can predict future suicide attempts through mediation by depression [23, 24]. Similarly, Madjar and his colleagues’ study has revealed that depression symptoms play a mediating role between adolescents’ sense of loneliness at school and NSSI behaviors [25].

Previous studies have demonstrated the negative impact of workplace violence on both physical and mental health. Participants who reported being bullied in the 30 days prior to the survey or having been in fights or sustained injuries within the past 12 months were found to be more likely to experience loneliness, with the likelihood of loneliness increasing in relation to the severity and frequency of bullying [26]. Additionally, numerous studies provide strong evidence of a significant relationship between workplace violence and depressive symptoms, as reported by Hsieh et al. [27] and Roche, Diers, Duffield, & Catling-Paull [28]. In fact, it was found that 36.3% of workplace violence victims experienced intermediate depressive symptoms, with 16% probably developing major depression [29]. In this context, several studies have confirmed the critical role of bullying behavior in predicting non-suicidal self-injury among children and adolescents [30]. Therefore, it is crucial for hospital administrator to implement effective strategies to prevent and address workplace violence to promote the mental health and well-being of nurse staff.

**Fig. 1:**
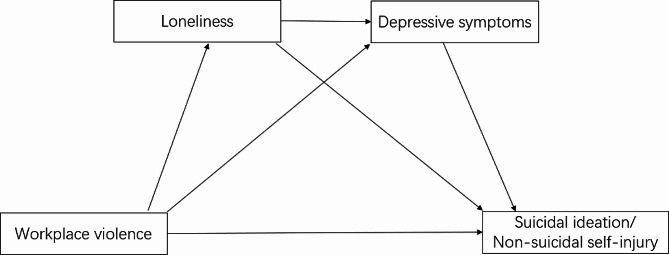
The conceptual model for whole sample, basing on previous study

In light of the significant implications of workplace violence, loneliness, and depressive symptoms on nurses’ suicidal ideation/NSSI, the Interpersonal-Psychological Theory of Suicide (IPTS) offers a comprehensive framework for elucidating the underlying mechanisms [31]. According to IPTS, suicidal behavior results from the interplay between the desire to die and the capability for suicide, with workplace violence potentially augmenting both components. Chronic exposure to violence heightens the desire to die by exacerbating feelings of loneliness and depression, while concurrently diminishing self-control mechanisms, thereby enhancing suicide capability. Despite the acknowledged significance of workplace violence, loneliness, depressive symptoms, and their collective impact on nurses’ mental health, significant gaps persist in understanding the underlying mechanisms. Existing research predominantly focuses on specific demographics, such as adolescents, with limited exploration within the nursing population. Addressing these lacunae is paramount, given the profound implications of NSSI and suicidal ideation on nurses’ well-being. Therefore, our research aims to investigate the mechanism by how workplace violence affects suicidal ideation or NSSI in nurse staff and to examine the mediating roles of loneliness and depressive symptoms, guided by the IPTS framework. The theoretical framework is as follows (Fig. 1).

## Methods

### Participants

This study was a cross-sectional design by convenient sampling methods. Participants were recruited from 18 local governmental hospitals of Dehong districts, Yunnan province, China, in July 2022. Participants can complete our survey by wenjuanxing software, which is biggest online questionnaires platform. Our inclusion criteria are: (1) Works in 18 local governmental hospitals; (2) Were not practice nurse; (3) Volunteered this survey and provided written informed consent. Our trained investigator fully interpreted the aim of this survey for each participants. With the help of nursing department of each hospital (distributing our questionnaire links), a total of 1965 nurse staffs were involved in this survey and 1774 questionnaires were completed, with a response of 90.3%. This study was approved by the Ethics Committee of Dehong people’s hospital (Code: DYLL-KY032).

The methodology employed the cross-sectional survey formula to determine the sample size, defined as follows:

$${\rm{N}} = \frac{{z_{1 - \partial /2}^2 \times pq}}{{{d^2}}}$$

Z_1−α/2_ represents the critical value for significance testing, with α set at 0.05, corresponding to 1.96. The variable p denotes the prevalence rate of psychological health issues, while q is its complement (q = 1-p). The parameter d signifies the permissible error, where d is set at 0.2p. Previous research indicates a spectrum of prevalence rates for suicidal ideation or NSSI problems among nurses, spanning from 9.1 to 10.8% [4, 32–34]. For this investigation, the conservative estimate of 9.1% was adopted for computations, necessitating a minimum sample size of 1267 participants, factoring in a non-response rate of 25%.

### Measures

#### Socio-demographic variables

Basic socio-demographic variables were collected including: age, sex, ethnic, marital status, residence, monthly income, educational level and work experience (years).

#### Workplace violence

The Chinese version of workplace violence scale (WVS) was used to assess workplace violence in this study [35]. WVS consists of five dimensions (PA: physical assault, EA: emotional abuse, T: threats, VSH: verbal sexual harassment, SA: sexual abuse) and each dimension were evaluated by a self-report item (e.g., EA: Have you encountered the emotional abuse from patients or patients’ relatives in the past years? Including cursing, disrespect, and disparagement words). Each item can been respond to zero times (scored 0), one time (scored 1), two or three times (scored 2), more than three times (scored 3). Higher sum score indicated severe level of workplace violence. This scale have been confirmed good validity and reliability in China [36], with the Cronbach’s α = 0.76 in this study.

#### Loneliness

Three-item loneliness scale was used to assess loneliness [37]. It is consists of three items (e.g., how often do you feel isolated from others? ), with response of hardly ever (scored 1), some of the time (scored 2), often (scored 3). A total score of this scale ranged from 3 to 9, with higher sum scores indicating severe level of loneliness. This scale have been used in the Chinese nurse population [38, 39]. The Cronbach’s α = 0.83 in this study.

#### Depressive symptoms

Depressive symptoms was assessed by the 9-item Patient Health Questionnaire (PHQ-9) [40]. This scale was consist of 9-item with response of “not at all” (scored 0), “several days” (scored 1), “more than half the days” (scored 2), and “nearly every day” (scored 3). The total scores was calculated each item and ranged from 0 to 27. Higher total scores indicated severe depressive symptoms. It have been confirmed good validity and reliability in Chinese nurse population [41–43], with the Cronbach’s α = 0.91 in this study.

#### Suicidal ideation and non-suicidal self-injury

Suicidal ideation was assessed by the single-item “During the past 12 months, did you have consider attempting suicide?” [5], and the response to this question was “none” (scored 0), “little” (scored 1), “sometimes” (scored 2), “often” (scored 3), and “always” (scored 4). Also, non-suicidal self-injury was assessed by the item “During the past 12 months, did you have non-suicidal self-injury”, and participants can been responded from “none” (scored 0) to “always” (scored 4) [4]. Any score other than zero was considered indicative of suicidal ideation/NSSI in our study. It has been widely applied among the nursing population in China [4, 5, 44, 45].

#### Statistical analysis

Descriptive analysis were present that frequency and percentages were used for categorical variables, Mean and standard deviation (SD) were used for continuous variables (Skewed distribution data is described using percentiles). Spearman’s correlation analysis were used to examine the association between our key variables (workplace violence, loneliness, depressive symptoms, suicidal ideation, and NSSI). After that, we further performed structural equation model (SEM) to explore the mechanism of workplace violence impact on suicidal ideation or NSSI via loneliness and depressive symptoms. In the details of the theoretical framework, workplace violence directly influences suicidal ideation/NSSI. Additionally, it leads to loneliness, which in turn triggers suicidal ideation/NSSI. Moreover, workplace violence causes depressive symptoms, contributing to suicidal ideation/NSSI. Furthermore, loneliness induces depressive symptoms, ultimately resulting in suicidal ideation/NSSI.

The goodness of model fit index was used to evaluate the quality of SEM model basing on Comparative fit index (CFI): measures goodness of fit of the hypothesized model compared to a baseline model, Tucker Lewis index (TLI): assesses the improvement in fit of the hypothesized model relative to a model assuming independence among variables, Normed fit index (NFI): indicates the improvement in fit of the hypothesized model over a null model where all variables are assumed independent., Relative fit index (RFI): quantifies the relative improvement in fit of the hypothesized model compared to a baseline model with no relationships, Root mean square error of approximation (RMSEA): a measure of lack of fit in the population with an adjustment for the parsimony of the model, Standardized Root Mean Squared Residual (SRMR): reflects the standardized square root mean of the residuals, assessing the absolute fit of the model, and CMIN/DF: evaluates the complexity of the model and the fit to the data by comparing the chi-square statistic to the degrees of freedom. Satisfactory goodness of fit was defined as CMIN/DF < 5, NFI > 0.90, CFI > 0.90, TLI > 0.90, RFI > 0.90, RMSEA < 0.08 and SRMR < 0.05 [46]. Bootstrap methods was performed to examine the significance of the total effect and indirect effect. In addition, our SEM analysis controlled for confounding factors such as age and sex, based on previous studies [47, 48]. We also found that other socio-demographic variables including marital status and ethnicity were significantly related to our key variable, as indicated by the ANOVA analysis. However, no significant relationships were observed for other variables. Therefore, in this study, age, sex, marital status, and ethnicity were controlled for in the analysis. All statistical analysis were conducted by SPSS 21.0, and SEM model were performed by SPSS amos software. The significance were set at *P* < 0.05 (two - tailed) in this study.

## Result

In this study, rigorous procedural controls were implemented to ensure methodological integrity throughout the testing phase. These controls encompassed the utilization of anonymous questionnaire measures, standardized testing protocols, and other established methodologies. Furthermore, to address potential common method biases, the Harman single-factor test was employed [49]. The results of the Harman single-factor test revealed the presence of four factors with characteristic roots exceeding 1. Notably, the variance explained by the primary factor amounted to 35.12%, falling below the critical threshold of 40%. This outcome signifies the absence of common method bias within the study’s findings.

As shown in Table 1, a total sample of 1774 nurse staff (1666 females and 108 males) were analyzed in this study, with a mean age of 32.00 (SD = 7.99) years. 1276 (71.9%) nurse staff were Han ethnics and 1200 (67.6%) were married. Most of nurse staff were married and 782 (44.1%) nurse staff earn 3001–5000 yuan monthly income. In addition, nurse staff 165 (7.8%) were reported different level of suicidal ideation and 139 (7.8%) participants were reported different level of non-suicidal self-injury.

**Table 1:** Socio-demographic characteristics and key variables outcomes of participants (*N* = 1774)

Characteristic	Number	Percent (%)
Age (years)	32.00 ± 7.99	
20–24	185	10.4
25–29	674	38.0
30–34	452	25.5
35–39	171	9.6
40–59	292	16.5
Sex		
Women	1666	93.9
Men	108	6.1
Ethnic		
Han	1276	71.9
Others	498	28.1
Marital status		
Unmarried	517	29.1
Married	1200	67.6
Divorce/others	57	3.2
Residence		
Rural	1071	60.4
Urban	703	39.6
Education level		
High school or lower	614	34.6
Bachelor’s degree or above	1160	65.4
Income (monthly)		
3000 or lower	498	28.1
3001–5000	782	44.1
5001–7000	325	18.3
7000 or higher	169	9.5
Experience (years)	10.83 ± 8.55	
0–4	393	22.2
5–9	592	33.4
10–14	391	22.0
15–19	128	7.2
20–40	270	15.2
Suicidal ideation		
Yes	165	9.3
No	1609	90.7
Non-suicidal self-injury		
Yes	139	7.8
No	1635	92.2
Key variables (Mean ± SD) Or median (P25, P75)		
Workplace violence	0.89 ± 1,81 0 (0, 1.00)	
Loneliness	5.26 ± 1.55	
Depressive symptoms	7.42 ± 5.13	
Suicidal ideation	1.13 ± 0.44	
Non-suicidal self-injury	1.11 ± 0.43	

Table 2 present that the basic characteristics of our key variables, the mean score of workplace violence, loneliness, depressive symptoms, suicidal ideation, and non-suicidal self-injury were 0.89 (SD = 1.81) (M_edian_ = 0, P_25%_ = 0, P_75%_ = 1), 5.26 (SD = 1.55), 7.42 (SD = 5.13), 1.13(SD = 0.44), 1.11(SD = 0.43), respectively. And spearman’s correlation further revealed that workplace violence, loneliness, depressive symptoms, suicidal ideation, and NSSI was positively associated each other.

**Table 2:** Correlation coefficient of key variables using Spearman correlation

Variables	1	2	3	4	5
1. Workplace violence	1				
2. Loneliness	0.163^***^	1			
3. Depressive symptoms	0.286^***^	0.487^***^	1		
4. Suicidal ideation	0.150^***^	0.263^***^	0.354^**^	1	
5. non-suicidal self-injury	0.191^***^	0.195^***^	0.291^***^	0.537^***^	1

Note: ^***^P < 0.001

**Fig. 2:**
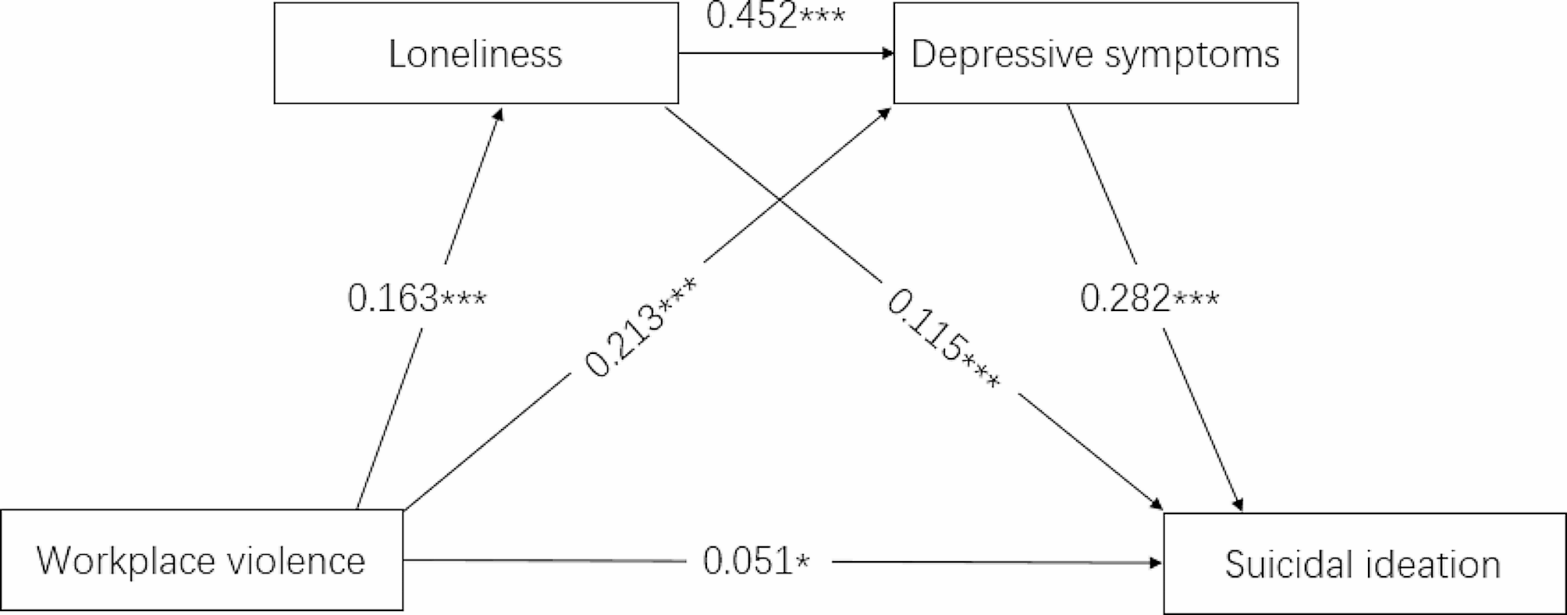
Association between workplace violence, loneliness, depression symptom and suicidal ideation and its mediation role of loneliness and depressive symptoms. (CMIN/DF = 3.482 NFI = 0.969 CFI = 0.977 TLI = 0.955 RFI = 0.938, RMSEA = 0.037 SRMR = 0.035) Note: results were shown as the standardized β value and age, sex, marital status, and ethnicity were adjusted in the models; ^***^ was represented for *P* < 0.001.

**Fig. 3:**
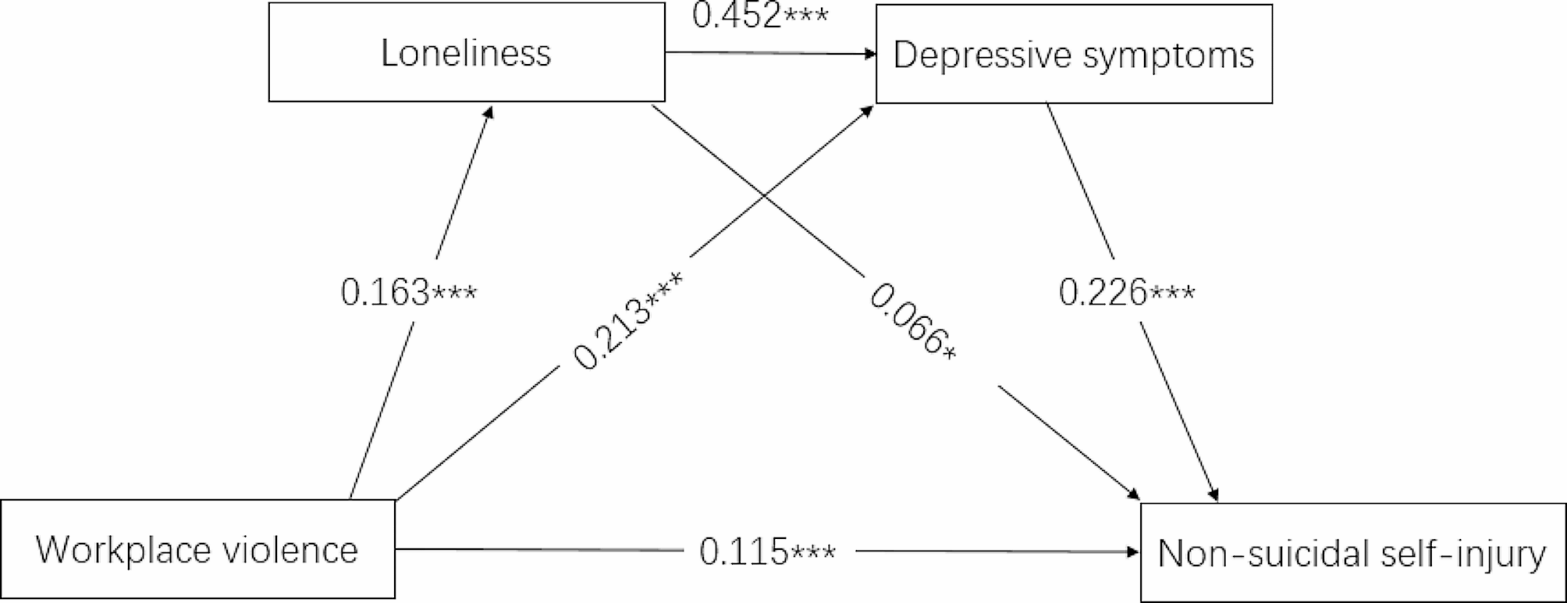
Association between workplace violence, loneliness, depression symptom and non-suicidal self-injury and its mediation role of loneliness and depressive symptoms. (CMIN/DF = 3.482 NFI = 0.967 CFI = 0.976 TLI = 0.953 RFI = 0.935, RMSEA = 0.037 SRMR = 0.034) Note: results were shown as the standardized β value and age, sex, marital status, and ethnicity were adjusted in the models; ^***^ was represented for *P* < 0.001.

The result of structural equation model analysis is present in Figs. 2 and 3. The final model of suicidal ideation shown as in Fig. 2. The final model showed a good model fit index: CMIN/DF = 3.482 NFI = 0.969 CFI = 0.977 TLI = 0.955 RFI = 0.938, RMSEA = 0.037 SRMR = 0.035. After controlling sex, age, marital status and ethnicity, the pathway effect of our key variables were are as follows: workplace violence to loneliness (β = 0.163, *P* < 0.001), workplace violence to depressive symptoms (β = 0.213, *P* < 0.001), workplace violence to suicidal ideation (β = 0.051, *P* < 0.05), loneliness to suicidal ideation (β = 0.115, *P* < 0.001), loneliness to depressive symptoms (β = 0.452, *P* < 0.001), depressive symptoms to suicidal ideation (β = 0.282, *P* < 0.001). In addition, bootstrap methods present that the total effect of workplace violence on suicidal ideation were 0.151 (95%CI = 0.090, 0.203), and the indirect effect of workplace violence on suicidal ideation via loneliness and depressive symptoms were 0.100 (95%CI = 0.085, 0.121), the indirect effect of loneliness on suicidal ideation via depressive symptoms were 0.128 (95%CI = 0.100, 0.158) (Table 3).

**Table 3:** Standardized total effect and indirect effect of study variables on Suicidal ideation or non-suicidal self-injury basing on bootstrap methods

Variables	Suicidal ideation		Non-suicidal self-injury	
	Indirect effect β (95%CI)	Total effect β (95%CI)	Indirect effect β (95%CI)	Total effect β (95%CI)
Workplace violence	0.100 (0.085, 0.121)	0.151 (0.090, 0.203)	0.075 (0.055, 0.096)	0.190 (0.123, 0.255)
Loneliness	0.128 (0.100, 0.158)	0.243 (0.205, 0.289)	0.102 (0.076, 0.130)	0.168 (0.124, 0.206)
Depressive symptoms	-	0.282 (0.227, 0.339)	-	0.226 (0.164, 0.280)

The final model of workplace violence on NSSI via loneliness and depressive symptoms was showed in Fig. 3. The final model shown a good model fit index: CMIN/DF = 3.482 NFI = 0.967 CFI = 0.976 TLI = 0.953 RFI = 0.935, RMSEA = 0.037 SRMR = 0.034. After control confounding factors sex, age, marital status and ethnicity, the specific pathway effect of key variable were: workplace violence to loneliness (β = 0.163, *P* < 0.001), workplace violence to depressive symptoms (β = 0.213, *P* < 0.001), workplace violence to NSSI (β = 0.115, *P* < 0.001), loneliness to NSSI (β = 0.066, *P* < 0.05), loneliness to depressive symptoms (β = 0.452, *P* < 0.001), depressive symptoms to NSSI (β = 0.226, *P* < 0.001). In addition, bootstrap methods present that the total effect of workplace violence on NSSI were 0.190 (95%CI = 0.123, 0.255), and the indirect effect of workplace violence on NSSI via loneliness and depressive symptoms were 0.075 (95%CI = 0.055, 0.096), the indirect effect of loneliness on NSSI via depressive symptoms were 0.102 (95%CI = 0.076, 0.130) (Table 3).

## Discussion

In this study, we revealed the mechanism of workplace violence on suicidal ideation/NSSI via loneliness and depressive symptoms. Finding of this study showed that loneliness and depressive symptoms plays a partial mediation role in the association between workplace violence and suicidal ideation/NSSI. Additionally, this study sheds light on the prevalence of suicidal ideation (9.3%) and NSSI (7.8%) among nurse staff in mainland China. And our rate of suicidal ideation exceeds the lifetime prevalence of 3.9% reported in a meta-analysis for the general Chinese population [50]. Additionally, our rate of non-suicidal self-injury (NSSI) surpasses the proportion of individuals who reported ever engaging in NSSI, which was 6.0% [51]. To the best of our knowledge, there have been limited investigations conducted globally, including China, that center on the topic of suicidal ideation or NSSI among nurses. In light of our findings revealing a prevalence of suicidal ideation and NSSI among nurses in China that surpasses that of the general population, it becomes imperative to delve into the public health ramifications of these results, emphasizing the urgent need for targeted interventions and policy measures to address the unique mental health challenges faced by this essential workforce, thereby bolstering their well-being and consequently elevating the quality of healthcare services delivered. In clinical settings, healthcare institutions should fortify mental health education and psychological counseling services for nurses, ensuring they receive requisite psychological support. At the policy level, governmental bodies and relevant authorities ought to devise mental health promotion policies specifically tailored to the nursing workforce, thereby ameliorating their mental health status and consequently enhancing the overall quality of healthcare services.

Our research demonstrated that depressive symptoms mediate the association between loneliness and suicidal ideation/NSSI. Yang’s study similarly found that depressive symptoms partially mediate the relationship between loneliness and suicidal ideation in nursing home residents [23]. Unsatisfied internal needs and an increasing sense of burden can harm seniors’ self-esteem, leading to feelings of hopelessness and a surge in depressive symptoms, which can in turn influence suicidal ideation [23]. Similarly, nurses who experience loneliness may have a reduced sense of belonging, indirectly triggering depression and further impacting suicidal ideation and NSSI [52].

Moreover, our findings indicate that loneliness and depressive symptoms partially mediate the link between workplace violence and suicidal ideation or NSSI. This finding is consistent with prior research indicating that depressive symptoms serve as a mediator, either partially or fully, in the relationship between conventional victimization and suicidal ideation [53–55]. For instance, Sampasa-Kanyinga et al. observed that depressive symptoms fully mediate the association between cyber victimization and suicidal ideation, plans, and attempts [56]. The indirect pathways through depression suggest that experiencing victimization may heighten adolescents’ susceptibility to depressive symptoms, subsequently escalating the risk of suicidal ideation [56]. Additionally, research by Md. Mehedi Hasan indicates that loneliness can act as a mediator in the relationship between bullying and suicidal ideation [57]. According to stress-diathesis theory [58], experiencing workplace violence, such as encounters with hostile patients, can exacerbate nurses’ sense of loneliness and helplessness, both towards themselves and their future prospects. This, in turn, may activate biological and cognitive vulnerabilities, including a predisposition to depressive symptoms [59]. Regrettably, once these negative emotions become internalized and entrenched, they may interact with environmental stressors, such as repeated victimization and secondary harm from others, further amplifying depressive symptoms [55]. As depression progresses, nurses may perceive their environment as unpredictable, uncontrollable, and intolerable, leading to emotional distress and potentially culminating in extreme behaviors such as suicidal ideation or non-suicidal self-injury.

Owing to the importance of NSSI and suicidal ideation on nurse’ health, addressing limitations in previous studies is essential to not just gain a fuller understanding of how workplace violence, loneliness, depressive symptoms, suicidal ideation and NSSI interrelate, but also for policymaker developing to implement prevention and intervention of NSSI and suicidal ideation. According to recent evidence, the majority of healthcare workers who have faced workplace violence do not report the incident. This could be due to their perception that reporting is pointless, or because they lack knowledge of whom to report to [60]. Our study highlights the need for comprehensive screening and intervention programs in healthcare settings, including those aimed at reducing workplace violence and addressing mental health issues such as loneliness and depressive symptoms. It is recommended that hospitals provide professional psychological consultations and support from administrators and nurse managers to nurses who report experiences of workplace violence.

There are several limitations that need to be considered in this study. Firstly, one of the primary limitations of this study lies in its cross-sectional design, which precludes the establishment of causal relationships between variables. While this study offers valuable insights into the associations among variables, it cannot definitively establish causality. To mitigate this limitation, future longitudinal studies are recommended to explore the temporal sequence of events and changes in variables over time, facilitating a more precise evaluation of causality. Longitudinal studies, by tracking participants over an extended period and gathering data at multiple time points, can furnish stronger evidence for causal inferences and a deeper comprehension of the interplay among the variables under scrutiny. Secondly, participants were recruited through convenience sampling solely from a specific region in China, potentially constraining the generalizability of our findings to a nationally representative sample. In comparison with Wang’s study, notable disparities emerged, particularly concerning sex (χ²/df = 22.119, *p* < 0.05) and marital status (χ²/df = 70.569, *p* < 0.05) [61]. Consequently, future research endeavors should consider a more expansive sampling approach to enhance the generalizability and representativeness of our findings. Thirdly, it is noteworthy that our study did not account for the potential influence of confounding variables, such as job stress, social support, or coping strategies, on the relationship between workplace violence and suicidal ideation/NSSI. Future research endeavors should consider incorporating these variables to provide a more comprehensive understanding of the dynamics at play. Finally, potential reporting and recall biases might affect the accuracy of this study’s conclusions. Reporting bias could lead to underestimating suicidal ideation or non-suicidal self-injury among nurses due to reluctance to disclose. Meanwhile, recall bias could influence the reliability of nurses’ recollections of past events. To address these biases, future studies could employ more objective measurement methods, such as validating self-reported information using medical records. Longitudinal studies could also help mitigate recall bias.

## Conclusion

The present study has identified loneliness and depressive symptoms as partial mediators in the relationship between workplace violence and suicidal ideation/NSSI among nurses. These findings imply that interventions to prevent NSSI and suicidal ideation should extend beyond direct approaches, addressing workplace stressors and promoting social connectedness to enhance overall mental health and well-being. Building upon our findings, we propose avenues for future research aimed at enhancing nurse well-being and fostering safer work environments. Future endeavors may involve developing and evaluating targeted programs encompassing stress management techniques, conflict resolution training, and improved reporting mechanisms for workplace violence incidents. By investing in these prevention and intervention strategies, we can work towards reducing workplace violence occurrences and enhancing the mental health of nurses, thereby creating environments conducive to their well-being and professional fulfillment.

## Data Availability

The datasets generated during and/or analysed during the current study are available from the corresponding author on reasonable request.

## Abbreviations

WVS: Workplace Violence Scale
SI: Suicidal ideation
NSSI: non-suicidal self-injury
PHQ-9: the 9-item Patient Health Questionnaire
SEM: Structural equation model
CFI: Comparative fit index
TLI: Tucker Lewis index
NFI: Normed fit index
RFI: Relative fit index
RMSEA: Root mean square error of approximation
SRMR: Standardized Root Mean Squared Residual
